# Importance of Genetic Diversity Assessment in Crop Plants and Its Recent Advances: An Overview of Its Analytical Perspectives

**DOI:** 10.1155/2015/431487

**Published:** 2015-03-19

**Authors:** M. Govindaraj, M. Vetriventhan, M. Srinivasan

**Affiliations:** ^1^Centre for Plant Breeding and Genetics, Tamil Nadu Agricultural University, Coimbatore 641 003, India; ^2^International Crops Research Institute for the Semi-Arid Tropics, Patancheru, Telangana 502324, India; ^3^School of Life Science, Bharathidasan University, Tiruchirappalli 620 024, India

## Abstract

The importance of plant genetic diversity (PGD) is now being recognized as a specific area since exploding population with urbanization and decreasing cultivable lands are the critical factors contributing to food insecurity in developing world. Agricultural scientists realized that PGD can be captured and stored in the form of plant genetic resources (PGR) such as gene bank, DNA library, and so forth, in the biorepository which preserve genetic material for long period. However, conserved PGR must be utilized for crop improvement in order to meet future global challenges in relation to food and nutritional security. This paper comprehensively reviews four important areas; (i) the significance of plant genetic diversity (PGD) and PGR especially on agriculturally important crops (mostly field crops); (ii) risk associated with narrowing the genetic base of current commercial cultivars and climate change; (iii) analysis of existing PGD analytical methods in pregenomic and genomic era; and (iv) modern tools available for PGD analysis in postgenomic era. This discussion benefits the plant scientist community in order to use the new methods and technology for better and rapid assessment, for utilization of germplasm from gene banks to their applied breeding programs. With the advent of new biotechnological techniques, this process of genetic manipulation is now being accelerated and carried out with more precision (neglecting environmental effects) and fast-track manner than the classical breeding techniques. It is also to note that gene banks look into several issues in order to improve levels of germplasm distribution and its utilization, duplication of plant identity, and access to database, for prebreeding activities. Since plant breeding research and cultivar development are integral components of improving food production, therefore, availability of and access to diverse genetic sources will ensure that the global food production network becomes more sustainable. The pros and cons of the basic and advanced statistical tools available for measuring genetic diversity are briefly discussed and their source links (mostly) were provided to get easy access; thus, it improves the understanding of tools and its practical applicability to the researchers.

## 1. Introduction

**Figure 1:**
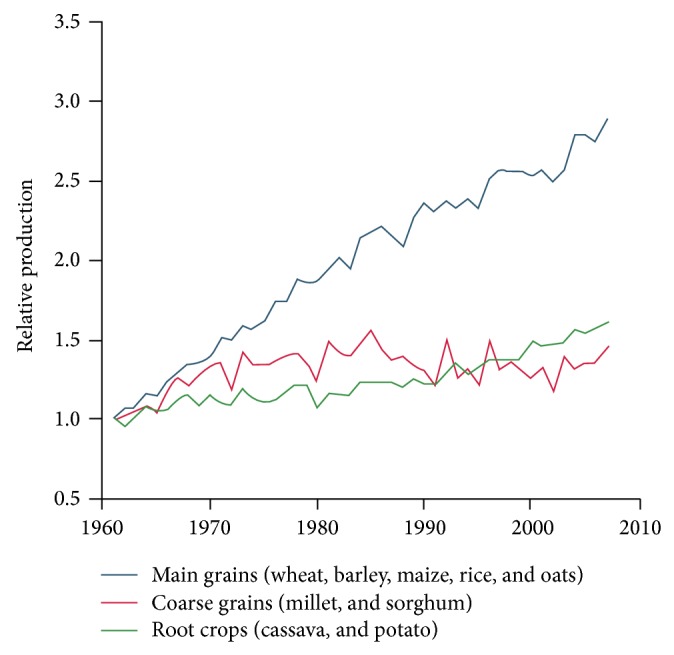
Changes in the relative global production of crops since 1961 (when relative production scaled to 1 (m.t) in 1961) (source: http://faostat.fao.org/default.aspx (2010)).

Diversity in plant genetic resources (PGR) provides opportunity for plant breeders to develop new and improved cultivars with desirable characteristics, which include both farmer-preferred traits (yield potential and large seed, etc.) and breeders preferred traits (pest and disease resistance and photosensitivity, etc.). From the very beginning of agriculture, natural genetic variability has been exploited within crop species to meet subsistence food requirement, and now it is being focused to surplus food for growing populations. In the middle of 1960s developing countries like India experienced the green revolution by meeting food demand with help of high-yielding and fertilizer responsive dwarf hybrids/varieties especially in wheat and rice (Figure 1). These prolonged activities that lead to the huge coverage of single genetic cultivars (boom) made situation again worse in other forms such as genetic erosion (loss of genetic diversity) and extinction of primitive and adaptive genes (loss of landraces). Today with an advancement of agricultural and allied science and technology, we still ask ourselves whether we can feed the world in 2050; this question was recently sensitized at the world food prize event in 2014 and remains that unanswered in every one hands since global population will exceed 9 billion in 2050. The per capita availability of food and water will become worse year after year coping with the undesirable climate change. Therefore, it becomes more important to look at the agriculture not only as a food-producing machine, but also as an important source of livelihood generation both in the farm and nonfarm sectors. Keeping the reservoir for cultivated and cultivable crops species is a principle for future agriculture, just like keeping a museum of cultural and spiritual specialty of diverse civilized humans in various geography for their historical evidence for future. The former can play a very important role in providing adaptive and productive genes, thus leading to long-term increases in food productivity which is further associated with environmental detriment. This paper will indicate the significance of genetic conservation and its analytical tools and techniques that are made widely available for utilization in postgenomic era. Plant and animal breeders introduced desirable genes and eliminated undesirable ones slowly, altering in the process of underlying heredity principle for several decades [1]. With the advent of new biotechnological tools and techniques, this process of genetic manipulation is being accelerated and it shortened the breeding cycles, and it can be carried out with more precision (neglecting environmental effects) and fast-track manner than the classical breeding techniques.

## 2. Significance of Genetic Conservation of Crop Plants

The growing population pressure and urbanization of agricultural lands and rapid modernization in every field of our day-to-day activities that create biodiversity are getting too eroded in direct and indirect way. For instance, land degradation, deforestation, urbanization, coastal development, and environmental stress are collectively leading to large-scale extinction of plant species especially agriculturally important food crops. On the other hand, system driven famine such as, Irish potato famine and Southern corn leaf blight epidemic in USA are the two instances of food crises caused by large-scale cultivation of genetically homogenous varieties of potato and corn, respectively. Even after these historical events, the importance of PGR had only got popular recognition when the spread of green revolution across cultivated crops threatened the conservation of land races [2]. Green revolution technologies introduced improved crop varieties that have higher yields, and it was hoped that they would increase farmers' income. Consequently, the Consultative Group of International Agricultural Researches (CIGAR) initiated gene banks and research centers of domestication for conserving PGR in most of the stable food crops around the world. Center for domestication: maize (Mexico), wheat and barley (middle/near East and North Africa), rice (North China), and potatoes (Peru); for further information see http://www.cigar.org/center/index.html.) The Food and Agriculture Organization (FAO) supported the International Treaty on Plant Genetic Resources (ITPGR) and UN supported the Convention on Biological Diversity (CBD) which are the international agreements that recognize the important role of genetic diversity conservation. Such treaty still plays in current and future food production as one of the major supremo [3].

Genetic diversity is the key pillar of biodiversity and diversity within species, between species, and of ecosystems (CBD, Article 2), which was defined at the Rio de Janeiro Earth Summit. However, the problem is that modern crop varieties, especially, have been developed primarily for high yielding potential under well endowed production conditions. Such varieties are often not suitable for low income farmers in marginal production environments as they are facing highly variable stress conditions [4]. Land races or traditional varieties have been found to have higher stability (adaptation over time) in low-input agriculture under marginal environments, thus, their cultivation may contribute farm level resilience in face of food production shocks [5, 6]. This is especially true in some part of Ethiopia where agroclimatic conditions are challenging, technological progress is slow, and market institutions are poorly developed and have no appropriate infrastructure [7, 8].

Why is genetic diversity important? The goal of conservation genetics is to maintain genetic diversity at many levels and to provide tools for population monitoring and assessment that can be used for conservation planning. Every individual is genetically unique by nature. Conservation efforts and related research are rarely directed towards individuals but genetic variation is always measured in individuals and this can only be estimated for collections of individuals in a population/species. It is possible to identify the genetic variation from phenotypic variation either by quantitative traits (traits that vary continuous and are governed by many genes, e.g., plant height) or discrete traits traits that fall into discrete categories and are governed by one or few major genes (e.g., white, pink, or red petal color in certain flowers) which are referred to as qualitative traits. Genetic variation can also be identified by examining variation at the level of enzymes using the process of protein electrophoresis. Further, genetic variations can also be examined by the order of nucleotides in the DNA sequence.

## 3. Erosion of Genetic Diversity due to Population Size: A Bottleneck Concept

It is well known that inbreeding is the most common phenomena in cross-pollinated crops, and in small outcross populations it has resulted in deleterious effects and loss of fitness of the population due to recombination between undesirable genes (recessive identical alleles). In natural population too, severe reductions in population size, the so-called* genetic bottleneck*, leads to loss of genetic diversity and increased susceptibility to infectious pests and diseases that supervene increased chances of extinction of an individual crop in question. Genetic models that predict the proportion of initial heterozygosity retained per generation is [1 − (1/2*N*_e_)] where *N*_e_ is the effective population size, usually less than *N*, the actual population size. Thus a population of *N*_e_ = 10 individuals loses 5% of its heterozygosity per generation. This indicates that severe bottlenecks degrade heterozygosity and genetic diversity [9]. Therefore, plant breeders have been advised to maintain the optimum population size for any trait conservation for specific purpose and its utilization for crop improvement. Thus, before quantifying the genetic diversity, it is essential to know the optimum population size and its representatives to ensure no biasness in diversity assessment that leads to wrong prediction of its value.

## 4. Climate Change and Its Impact on Plant Genetic Resources

The most profound and direct impacts of climate change over previous decade and the next few decades will surely be on agriculture and food security. The effects of climate change will also depend on current production conditions. The area where already being obstructed by other stresses, such as pollution and will likely to have more adverse impact by changing climate. Food production systems rely on highly selected cultivars under better endowed environments but it might be increasingly vulnerable to climate change impacts such as pest and disease spread. If food production levels decreases over the year, there will be huge pressure to cultivate the crops under marginal lands or implement unsustainable practices that, over the long-term, degrade lands and resources and adversely impact biodiversity on and near agricultural areas. In fact, such situations have already been experienced by most of the developing countries. These changes have been seen to cause a decrease in the variability of those genetic loci (alleles of a gene) controlling physical and phenotypic responses to changing climate [10]. Therefore, genetic variation holds the key to the ability of populations and species to persist over evolutionary period of time through changing environments [11]. If this persists, neither any organism can predict its future (and evolutionary theory does not require them to) nor can any of those organisms be optimally adapted for all environmental conditions. Nonetheless, the current genetic composition of a crop species influences how well its members will adapt to future physical and biotic environments.

The population can also migrate across the landscape over generations. By contrast, populations that have a narrow range of genotypes and are more phenotypically uniform may merely fail to survive and reproduce at all as the conditions become less locally favorable. Such populations are more likely to become extirpated (locally extinct), and in extreme cases the entire plant species may end up at risk of extinction. For example, the Florida Yew (*Torreya taxifolia*) is currently one of the rarest conifer species in North America. But in the early Holocene (10,000 years ago), when conditions in southeastern North America were cooler and wetter than today, the species was probably widespread. The reasons for that are not completely understood, but* T. taxifolia* failed to migrate towards the northward as climate changed during the Holocene. Today, it is restricted to a few locations in the Apalachicola River Basin in southern Georgia and the Florida panhandle. As the* T. taxifolia* story illustrates, once plant species are pushed into marginal habitat at the limitations of their physiological tolerance, they may enter an extinction vortex, a downward cycle of small populations, and so on [12, 13]. Reduced genetic variability is a key step in the extinction vortex. Gene banks must be better to respond to novel and increased demands on germplasm for adapting agriculture to climate change. Gene banks need to include different characteristics in their screening processes and their collections need to be comprehensive, including what are now considered minor crops, and that may come with huge impact on food baskets.

## 5. Assessment of Genetic Diversity in Crop Plants

The assessment of genetic diversity within and between plant populations is routinely performed using various techniques such as (i) morphological, (ii) biochemical characterization/evaluation (allozyme), in the pregenomic era, and (iii) DNA (or molecular) marker analysis especially single nucleotide polymorphism (SNPs) in postgenomic era. Markers can exhibit similar modes of inheritance, as we observe for any other traits, that is, dominant/recessive or codominant. If the genetic pattern of homozygotes can be distinguished from that of heterozygotes, then a marker is said to be codominant. Generally codominant markers are more informative than the dominant markers.

Morphological markers are based on visually accessible traits such as flower color, seed shape, growth habits, and pigmentation, and it does not require expensive technology but large tracts of land area are often required for these field experiments, making it possibly more expensive than molecular assessment in western (developed) countries and equally expensive in Asian and Middle East (developing) countries considering the labour cost and availability. These marker traits are often susceptible to phenotypic plasticity; conversely, this allows assessment of diversity in the presence of environmental variation which cannot be neglected from the genotypic variation. These types of markers are still having advantage and they are mandatory for distinguishing the adult plants from their genetic contamination in the field, for example, spiny seeds, bristled panicle, and flower/leaf color variants.

Second type of genetic marker is called biochemical markers, allelic variants of enzymes called isozymes that are detected by electrophoresis and specific staining. Isozyme markers are codominant in nature. They detect diversity at functional gene level and have simple inheritance. It requires only small amounts of plant material for its detection. However, only a limited number of enzymes markers are available and these enzymes are not alone but it has complex structural and special problems; thus, the resolution of genetic diversity is limited to explore.

The third and most widely used genetic marker type is molecular markers, comprising a large variety of DNA molecular markers, which can be employed for analysis of genetic and molecular variation. These markers can detect the variation that arises from deletion, duplication, inversion, and/or insertion in the chromosomes. Such markers themselves do not affect the phenotype of the traits of interest because they are located only near or linked to genes controlling the traits. These markers are inherited both in dominant and codominant patterns. Different markers have different genetic qualities (they can be dominant or codominant, can amplify anonymous or characterized loci, can contain expressed or nonexpressed sequences, etc.). A molecular marker can be defined as a genomic locus, detected through probe or specific starter (primer) which, in virtue of its presence, distinguishes unequivocally the chromosomic trait which it represents as well as the flanking regions at the 3′ and 5′ extremity [14]. Molecular markers may or may not correlate with phenotypic expression of a genomic trait. They offer numerous advantages over conventional, phenotype-based alternatives as they are stable and detectable in all tissues regardless of growth, differentiation, development, or defense status of the cell. Additionally, they are not confounded by environmental, pleiotropic, and epistatic effects. We are not describing much about the pregenomic era tools, since our paper deals with genomic advances and its assistance in crop genetic diversity assessment.

## 6. Analyses of Genetic Diversity in Genomic Era

A comprehensive study of the molecular genetic variation present in germplasm would be useful for determining whether morphologically based taxonomic classifications reveal patterns of genomic differentiation. This can also provide information on the population structure, allelic richness, and diversity parameters of germplasm to help breeders to use genetic resources with less prebreeding activities for cultivar development more effectively. Now germplasm characterization based on molecular markers has gained importance due to the speedy and quality of data generated. For the readers benefit, the availability of different DNA markers acronyms is given in Abbreviations section.

### 6.1. Molecular Markers

DNA (or molecular) markers are the most widely used type of marker predominantly due to their abundance. They arise from different classes of DNA mutations such as substitution mutations (point mutations), rearrangements (insertions or deletions), or errors in replication of tandemly repeated DNA [15]. These markers are selectively neutral because they are usually located in noncoding regions of DNA in a chromosome. Unlike other markers, DNA markers are unlimited in number and are not affected by environmental factors and/or the developmental stage of the plant [16]. DNA markers have numerous applications in plant breeding such as (i) marker assisted evaluation of breeding materials like assessing the level of genetic diversity, parental selection, cultivar identity and assessment of cultivar purity [16–26], study of heterosis, and identification of genomic regions under selection, (ii) marker assisted backcrossing, and (iii) marker assisted pyramiding [27].

Molecular markers may be broadly divided into three classes based on the method of their detection: hybridization-based, polymerase chain reaction- (PCR-) based, and DNA sequence-based. Restriction fragment length polymorphisms (RFLPs) are hybridization-based markers developed first in human-based genetic study during 1980s [28, 29] and later they were used in plant research [30]. RFLP is based on the variation(s) in the length of DNA fragments produced by a digestion of genomic DNAs and hybridization to specific markers of two or more individuals of a species is compared. RFLPs have been used extensively to compare genomes in the major cereal families such as rye, wheat, maize, sorghum, barley, and rice [31–33]. The advantages of RFLPs include detecting unlimited number of loci and being codominant, robust, and reliable and results are transferable across populations. However, RFLPs are highly expensive, time consuming, labour intensive, larger amounts of DNA required, limited polymorphism especially in closely related lines [34]. At present polymerase chain reaction- (PCR-) based marker systems are more rapid and require less plant material for DNA extraction. Rapid amplified polymorphic DNAs (RAPDs) were the first of PCR-based markers and are produced by PCR machines using genomic DNA and arbitrary (random) primers which act as both forward and backward primers in creation of multiple copies of DNA strands [35, 36]. The advantages of RAPDs include being quick and simple and inexpensive and the facts that multiple loci from a single primer are possible and a small amount of DNA is required. However, the results from RAPDs may not be reproduced in different laboratories and only can detect the dominant traits of interest [34]. Amplified fragment length polymorphisms (AFLPs) combine both PCR and RFLP [37]. AFLP is generated by digestion of PCR amplified fragments using specific restriction enzymes that cut DNA at or near specific recognition site in nucleotide sequence. AFLPs are highly reproducible and this enables rapid generation and high frequency of identifiable AFLPs, making it an attractive technique for identifying polymorphisms and for determining linkages by analyzing individuals from a segregating population [37]. Another class of molecular markers which depends on the availability of short oligonucleotide repeats sequences in the genome of plants such as SSR, STS, SCAR, EST-SSR, and SNP. Many authors reviewed in detail different markers techniques [38, 39]. In this paper we are presenting the most widely used molecular markers and next generation sequencing technologies in detail in the following section.

### 6.2. Simple Sequence Repeat or Microsatellite

Microsatellites [40] are also known as simple sequence repeats (SSRs), short tandem repeats (STRs), or simple sequence length polymorphisms (SSLPs) which are short tandem repeats, their length being 1 to 10 bp. Some of the literatures define microsatellites as 2–8 bp [41], 1–6 bp [42], or even 1–5 pb repeats [43]. SSRs are highly variable and evenly distributed throughout the genome and common in eukaryotes, their number of repeated units varying widely among crop species. The repeated sequence is often simple, consisting of two, three, or four nucleotides (di-, tri-, and tetranucleotide repeats, resp.). One common example of a microsatellite is a dinucleotide repeat (CA)*n*, where *n* refers to the total number of repeats that ranges between 10 and 100. These markers often present high levels of inter- and intraspecific polymorphism, particularly when tandem repeats number is 10 or greater [44]. PCR reactions for SSRs are performed in the presence of forward and reverse primers that anneal at the 5′ and 3′ ends of the template DNA, respectively. These polymorphisms are identified by constructing PCR primers for the DNA flanking the microsatellite region. The flanking regions tend to be conserved within the species, although sometimes they may also be conserved in higher taxonomic levels.

PCR fragments are usually separated on polyacrylamide gels in combination with AgNO_3_ staining, autoradiography, or fluorescent detection systems. Agarose gels (usually 3%) with ethidium bromide (EBr) can also be used when differences in allele size among samples are larger than 10 bp. However, the establishment of microsatellite primers from scratch for a new species presents a considerable technical challenge. Several protocols have been developed [43, 45–47] and details of the methodologies are reviewed by many authors [48–50]. The loci identified are usually multiallelic and codominant. Bands can be scored either in a codominant or as present or absent. The microsatellite-derived primers can often be used with many varieties and even other species because the flanking DNA is more likely to be conserved. These required markers are evenly distributed throughout the genome, easily automated, and highly polymorphic and have good analytic resolution and high reproducibility making them a preferred choice of markers [51], most widely used for individual genotyping, germplasm evaluation, genetic diversity studies, genome mapping, and phylogenetic and evolutionary studies. However, the development of microsatellites requires extensive knowledge of DNA sequences, and sometimes they underestimate genetic structure measurements; hence they have been developed primarily for agricultural species, rather than wild species [39].

### 6.3. EST-SSRs

An alternative source of SSRs development is development of expressed sequence tag- (EST-) based SSRs using EST databases has been utilized [52–58]. With the availability of large numbers of ESTs and other DNA sequence data, development of EST-based SSR markers through data mining has become fast, efficient, and relatively inexpensive compared with the development of genomic SSRs [59]. This is due to the fact that the time-consuming and expensive processes of generating genomic libraries and sequencing of large numbers of clones for finding the SSR containing DNA regions are not needed in this approach [60]. However, the development of EST-SSRs is limited to species for which this type of database exists as well as being reported to have lower rate of polymorphism compared to the SSR markers derived from genomic libraries [61–64].

### 6.4. Single Nucleotide Polymorphisms (SNPs)

Single nucleotide polymorphisms (SNPs) are DNA sequence variations that occur when a single nucleotide (A, T, C, or G) in the genome sequence is changed, that is, single nucleotide variations in genome sequence of individuals of a population. These polymorphisms are single-base substitutions between sequences. SNPs occur more frequently than any other type of markers and are very near to or even within the gene of interest. SNPs are the most abundant in the genomes of the majority of organisms, including plants, and are widely dispersed throughout genomes with a variable distribution among species. SNPs can be identified by using either microarrays or DHPLC (denaturing high-performance liquid chromatography) machines. They are used for a wide range of purposes, including rapid identification of crop cultivars and construction of ultrahigh-density genetic maps. They provide valuable markers for the study of agronomic or adaptive traits in plant species, using strategies based on genetic mapping or association genetics studies.

### 6.5. Diversity Arrays Technology (DArT)

A DArT marker is a segment of genomic DNA, the presence of which is polymorphic in a defined genomic representation. A DArT was developed to provide a practical and cost-effective whole genome fingerprinting tool. This method provides high throughput and low cost data production. It is independent from DNA sequence; that is, the discovery of polymorphic DArT markers and their scoring in subsequent analysis does not require any DNA sequence data. The detail of methodology for DArT is described by Jaccoud et al. [65] and Semagn et al. [38] as well as in website http://www.diversityarrays.com/.

To identify the polymorphic markers, a complexity reduction method is applied on the metagenome, a pool of genomes representing the germplasm of interest. The genomic representation obtained from this pool is then cloned and individual inserts are arrayed on a microarray resulting in a “discovery array.” Labelled genomic representations prepared from the individual genomes included in the pool are hybridized to the discovery array. Polymorphic clones (DArT markers) show variable hybridization signal intensities for different individuals. These clones are subsequently assembled into a “genotyping array” for routine genotyping. DArT is one of the recently developed molecular techniques and it has been used in rice [66], wheat [38, 67, 68], barley [69], eucalyptus [70],* Arabidopsis* [71], cassava [72], pigeon-pea [73], and so forth.

DArT markers can be used as any other genetic marker. With DArT, comprehensive genome profiles are becoming affordable regardless of the molecular information available for the crop. DArT genome profiles are very useful for characterization of germplasm collections, QTL mapping, reliable and precise phenotyping, and so forth. However, DArT technique involves several steps, including preparation of genomic representation for the target species, cloning, data management, and analysis, requiring dedicated software such as DArTsoft and DArTdb. DArT markers are primarily dominant (present or absent) or differences in intensity, which limits its value in some application [38].

## 7. Next Generation Sequencing

DNA sequencing is the determination of the order of the nucleotide bases, A (adenine), G (guanine), C (cytosine), and T (thymine), present in a target molecule of DNA. DNA sequencing technology has played a pivotal role in the advancement of molecular biology [74]. Next generation sequencing (NGS) or second generation sequencing technologies are revolutionizing the study of variation among individuals in a population. Most NGS technologies reduce the cost and time required for sequencing than Sanger-style sequencing machines (first generation sequencing). The following is the list of NGS technologies available at present, namely, the Roche/454 FLX, the Illumina/Solexa Genome Analyzer, the Applied Biosystems SOLiD System, the Helicos single-molecule sequencing, and pacific Biosciences SMRT instruments. These techniques have made it possible to conduct robust population-genetic studies based on complete genomes rather than just short sequences of a single gene.

The Roche/454 FLX, based on sequencing-by-synthesis with pyrophosphate chemistry, was developed by 454 Life Sciences and was the first next generation sequencing platform available on the market [75]. The Solexa sequencing platform was commercialized in 2006. The working principle is sequencing-by-synthesis chemistry. The Life Technologies SOLiD system is based on a sequencing-by-ligation technology. This platform has its origins in the system described by Shendure et al. [76] and in work by McKernan et al. [77] at Agencourt Personal Genomics (acquired by Applied Biosystems in 2006). Helicos true single molecule sequencing (tSMS) technology is an entirely novel approach to DNA sequencing and genetic analysis and offers significant advantages over both traditional and “next generation” sequencing technologies. Helicos offers the first universal genetic analysis platform that does not require amplification. Pursuing a single molecule sequencing strategy simplifies the DNA sample preparation process, avoids PCR-induced bias and errors, simplifies data analysis, and tolerates degraded samples. Helicos single-molecule sequencing is often referred to as third generation sequencing. The detailed methodology, advantages, and disadvantages of each NGS technology were reviewed by many authors [78–81].

## 8. Analysis of Genetic Diversity from Molecular Data

It is essential to know the different ways that the data generated by molecular techniques can be analyzed before their application to diversity studies. Two main types of analysis are generally followed: (i) analysis of genetic relationships among samples and (ii) calculation of population genetics parameters (in particular diversity and its partitioning at different levels). The analysis of genetic relationships among samples starts with the construction of a matrix, sample × sample pair-wise genetic distance (or similarities).

The advent and explorations of molecular genetics led to a better definition of Euclidean distance to mean a quantitative measure of genetic difference calculated between individuals, populations, or species at DNA sequence level or allele frequency level. Genetic distance and/or similarity between two genotypes, populations, or individuals may be calculated by various statistical measures depending on the data set. The commonly used measures of genetic distance (GD) or genetic similarity (GS) are (i) Nei and Li's [82] coefficient (GD_NL_), (ii) Jaccard's [83] coefficient (GD_J_), (iii) simple matching coefficient (GD_SM_) [84], and (iv) modified Rogers' distance (GD_MR_). Genetic distance determined by the above measures can be estimated as follows:

(1) $$\begin{array}{c} \text{G}{\text{D}}_{\text{NL}}=1=\left[\frac{2N_{11}}{\left(2N_{11}+N_{10}+N_{01}\right)}\right],\\ {\text{GD}}_{\text{J}}=1=\left[\frac{N_{11}}{\left(N_{11}+N_{10}+N_{01}\right)}\right],\\ {\text{GD}}_{\text{SM}}=1=\left[\frac{\left(N_{11}+N_{00}\right)}{\left(N_{11}+N_{10}+N_{01}+N_{00}\right)}\right],\\ \text{G}{\text{D}}_{\text{MR}}={\left[\frac{\left(N_{10}+N_{01}\right)}{2N}\right]}^{0.5}, \end{array}$$

where *N*_11_ is the number of bands/alleles present in both individuals; *N*_00_ is number of bands/alleles absent in both individuals; *N*_10_ is the number of bands/alleles present only in the individual *i*; *N*_01_ is the number of bands/alleles present only in the individual *j*; and *N* represents the total number of bands/alleles. Readers are requested to read Mohammadi and Prasanna [85] review paper for more details about different GD measures.

There are two main ways of analyzing the resulting distance (or similarity) matrix, namely, principal coordinate analysis (PCA) and dendrogram (or clustering, tree diagram). PCA is used to produce a 2 or 3 dimensional scatter plot of the samples such that the distances among the samples in the plot reflect the genetic distances among them with a minimum of distortion. Another approach is to produce a dendrogram (or tree diagram), that is, grouping of samples together in clusters that are more genetically similar to each other than to samples in other clusters. Different algorithms were used for clustering, but some of the more widely used ones include unweighted pair group method with arithmetic averages (UPGMA), neighbour-joining method, and Ward's method [86].

The molecular data can be scored in presence/absence matrices manually or with the aid of specific software. However, because these techniques are based on the incorporation of genomic elements in the primer sets or else target specific regions in the genome, biases affecting the evaluation process can occur. Although many recently developed targeting methods detect large numbers of polymorphisms, not many studies to date have utilized them, largely due to their unfamiliarity. In many cases the drawbacks are unknown. These mainly affect the analysis of the banding patterns produced, largely depending on the nature of the methods and whether they generate dominant or codominant markers. We presented a brief description of common/basic statistical approaches and its principle with the pros and cons of each method for measuring genetic diversity and it is given in Table 1. These are self-explanatory; therefore, the features and method of calculations were not much discussed separately in our text.

## 9. Assessment of Genetic Diversity in Postgenomic Era

Many software programs are available for assessing genetic diversity; however, most of them are freely available through source link to internet and corresponding institute web links are given in Table 2. In this section, we described some of the programs available which are mostly used in molecular diversity analyses in the postgenomic era (Table 2). Many of these perform similar tasks, with the main differences being in the user interface, type of data input and output, and platform. Thus, choosing which to use depends heavily on individual preferences.

## 10. Conclusion

Agriculturist has been realized that diverse plant genetic resources are priceless assets for humankind which cannot be lost. Such materials increasingly required to accessible for feeding a burgeoning world population in future (>9 billion in 2050). Presence of genetic variability in crops is essential for its further improvement by providing options for the breeders to develop new varieties and hybrids. This can be achieved through phenotypic and molecular characterization of PGR. Sometimes, large size of germplasm may limit their use in breeding. This may be overcome by developing and using subsets like core and minicore collection representing the diversity of the entire collection of the species. Molecular markers are indispensable tools for measuring the diversity of plant species. Low assay cost, affordable hardware, throughput, convenience, and ease of assay development and automation are important factors when choosing a technology. Now with the high throughput molecular marker technologies ensuring speed and quality of data generated, it is possible to characterize the larger number of germplasm with limited time and resources. Next generation sequencing reduced the cost and time required for sequencing the whole genome. Many software packages are available for assessing phenotypic and molecular diversity parameters that increased the efficiency of germplasm curators and, plant breeders to speed up the crop improvement. Therefore, we believe that this paper provides useful and contemporary information at one place; thus, it improves the understanding of tools for graduate students and also practical applicability to the researchers.

## Figures and Tables

**Table 1:** Some basic statistical concept on genomic data for genetic diversity assessment.

Concept terms	Description/features	Formulae/pros/cons
Band-based approaches	Easiest way to analyze and measure diversity by focusing on presence or absence of banding pattern.	Routinely use individual level. Totally relay on marker type and polymorphism

(1) Measuring polymorphism	Observing the total number of polymorphic bands (PB) and then calculating the percentage of polymorphic bands.	This “band informativeness” (Ib) can be represented on a scale ranging from 0 to 1 according to the formula Ib = 1 − (2 × |0.5 − *p*|), where *p* is the portion of genotypes containing the band.

(2) Shannon's information index (*I*)	It is called the Shannon index of phenotypic diversity and is widely applied.	*I* = −∑*p*_*i*_log_2_⁡*p*_*i*_. These methods depend on the extraction of allelic frequencies.

(3) Similarity coefficients	Utilize similarity or dissimilarity (the inverse of the previous one) coefficients. The Jaccard coefficient (*J*) only takes into account the bands present in at least one of the two individuals. It is therefore unaffected by homoplasic absent bands (where the absence of the same band is due to different mutations). The simple-matching index (SM) maximizes the amount of information provided by the banding patterns considering all scored loci. The Neil and Li index (SD) doubles the weight for bands present in both individuals, thus giving more attention to similarity than dissimilarity.	(i) **Jaccard similarity coefficient **or Jaccard index *J* = *a*/(*a* + *b* + *c*). (ii) **Simple matching coefficient **or index SM *= *(*n* − *b* − *c*)/*n* . (iii) **Sørensen-Dice index **or Nei and Li index SD = 2*a*/2*a* + *b* + *c* where *a* is the number of bands (1 s) shared by both individuals; b is the number of positions where individual *i* has a band, but *j* does not; *c* is the number of positions where individual *j* has a band, but *i* does not; and *n* is the total number of bands (0 s and 1 s).

(4) Allele frequency based approaches	Measure variability by describing changes in allele frequencies for a particular trait over time, more population oriented than band-based approaches.	These methods depend on the extraction of allelic frequencies from the data. The accurate estimates of frequencies essentially influence the results of different indices calculated for further measurements of genetic diversity.

(5) Allelic diversity (*A*)	Easiest ways to measure genetic diversity is to quantify the number of alleles present. Allelic diversity (*A*) is the average number of alleles per locus and is used to describe genetic diversity.	*A* = *n*_i_/*n*_l_ where *n*_i_ is the total number of alleles over all loci; *n*_l_ is the number of loci. It is less sensitive to sample size and rare alleles and is calculated as *n*_*e*_ = 1/∑*p*_*i*_^2^ *p*_*i*_^2^ ability; it provides information about the dispersal ability of the organism and the degree of isolation among populations.

(6) Effective population size (*N*_e_)	It provides a measure of the rate of genetic drift, the rate of genetic diversity loss, and increase of inbreeding within a population.	Effective size of a population is an idealized number, since many calculations depend on the genetic parameters used and on the reference generation. Thus, a single population may have many different effective sizes which are biologically meaningful but distinct from each other.

(7) Heterozygosity (*H*)	There are two types of heterozygosity observed (*H*_O_) and expected (*H*_E_). The *H*_O_ is the portion of genes that are heterozygous in a population and *H*_E_ is estimated fraction of all individuals that would be heterozygous for any randomly chosen locus. Typically values for *H*_E_ and *H*_O_ range from 0 (no heterozygosity) to nearly 1 (a large number of equally frequent alleles). If *H*_O_ and *H*_E_ are similar (they do not differ significantly), mating in the populations is random. If *H*_O_ < *H*_E_, the population is inbreeding; if *H*_O_ > *H*_E_, the population has a mating system avoiding inbreeding.	Expected *H*_E_ is calculated based on the square root of the frequency of the null (recessive) allele as follows: *H*_E_ = 1 − ∑_*i*_^*n*^ *p*_*i*_^2^ where *p*_*i*_ is the frequency of the *i*th allele. *H*_O_ is calculated for each locus as the total number of heterozygotes divided by sample size.

(8) *F*-statistics	In population genetics the most widely applied measurements besides heterozygosity are *F*-statistics, or fixation indices, to measure the amount of allelic fixation by genetic drift. The *F*-statistics are related to heterozygosity and genetic drift. Since inbreeding increases the frequency of homozygotes, as a consequence, it decreases the frequency of heterozygotes and genetic diversity.	Three indexes can be calculated as follows: *F*_IT_ = 1 − (*H*_I_/*H*_T_), *F*_IS_ = 1 − (*H*_I_/*H*_S_), *F*_ST_ = 1 − (*H*_S_/*H*_T_), where *H*_I_ is the average *H*_O_ within each population, *H*_S_ is the average *H*_E_ of subpopulations assuming random mating within each population, and *H*_T_ is the *H*_E_ of the total population assuming random mating within subpopulations and no divergence of allele frequencies among subpopulations.

**Table 2:** List of analytical programs for measuring molecular (genetic) diversity.

Analytical tools	Data type	Main features	Source links	Reference
Arlequin	RFLPs, DNA sequences, SSR data, allele frequencies, or standard multilocus genotypes.	(i) Estimation allele and haplotype frequencies. (ii) Tests of departure from linkage equilibrium, departure from selective neutrality and demographic equilibrium. (iii) Estimation or parameters from past population expansions. (iv) Thorough analyses of population subdivision under the AMOVA framework and so forth. (v) Current version: Arlequin ver 3.5.1.3.	http://cmpg.unibe.ch/software/arlequin3	Schneider et al. [87] Excoffier et al. [88]

DnaSP	DNA sequence data	(i) Estimating several measures of DNA sequence variation within and between populations (in noncoding, synonymous, or nonsynonymous sites or in various sorts of codon positions), as well as linkage disequilibrium, recombination, gene flow, and gene conversion parameters. (ii) DnaSP can also carry out several tests of neutrality: Hudson et al. [89], Tajima [90], McDonald and Kreitman [46], Fu and Li [91], and Fu [92] tests. Additionally, DnaSP can estimate the confidence intervals of some test-statistics by the coalescent and so forth. (iii) Current version: DnaSP v5.10.01.	http://www.ub.edu/dnasp	J. Rozas and R. Rozas, [93–95] Librado and Rozas [96]

PowerMarker	SSR, SNP, and RFLP data	(i) Computes several summary statistics for each marker locus, including allele number, missing proportion, heterozygosity, gene diversity, polymorphism information content (PIC), and stepwise patterns for microsatellite data. (ii) PowerMarker is also used to compute allele frequency, genotype frequency, haplotype frequency for unrelated individuals, Hardy-Weinberg equilibrium, pairwise linkage disequilibrium, multilocus linkage disequilibrium, consensus trees, population structure, Mantel's test, triangle plotting and visualization of linkage disequilibrium results. (iii) Current version: PowerMarker V3.25.	http://statgen.ncsu.edu/powermarker/	Liu and Muse [97]

DARwin	Single data (for haploids, homozygote diploids, and dominant markers), allelic data, and sequence data	(i) Most widely used for various dissimilarity and distance estimations for different data, tree construction methods including hierarchical trees with various aggregation criteria (weighted or unweighted), Neighbor-Joining tree (weighted or unweighted), Scores method and principal coordinate analysis, and so forth. (ii) Current version: DARwin v5.0.156.	http://darwin.cirad.fr/darwin	Perrier and Jacquemoud-Collet [98]

NTSYSpc	Single data (for haploids, homozygote diploids, and dominant markers), allelic data, and sequence data	(i) Used for clustering analysis, ordination analysis, principal component analysis, principal coordinate analysis, scaling analysis, and comparison of two matrices (Mantel test, Mantel [99] and so forth). (ii) Current version: NTSYSpc version 2.2.	http://www.exetersoftware.com/cat/ntsyspc/ntsyspc.html	Rohlf [100]

MEGA	DNA sequence, protein sequence, evolutionary distance, or phylogenetic tree data	(i) Molecular evolutionary genetics analysis (MEGA) is most widely used for aligning sequences, estimating evolutionary distances, building tree from sequence data, testing tree reliability, and so forth. (ii) Current version: MEGA6.	http://www.megasoftware.net	Kumar et al. [101–103] Tamura et al. [104]

PAUP	Molecular sequences, morphological data, and other data types	(i) Used for inferring and interpreting phylogenetic trees using parsimony, distance matrix, invariants, maximum likelihood methods, and many indices and statistical analyses. (ii) Current version: PAUP version 4.0.	http://paup.csit.fsu.edu/	Swofford [105]

STRUCTURE	All types of markers including mostly used markers like SSRs, SNPs, RFLPs, dArT, and so forth.	(i) A free program to investigate population structure; it includes inferring the presence of distinct populations, assigning individuals to populations, studying hybrid zones, identifying migrants and admixed individuals, and estimating population allele frequencies in situations where many individuals are migrants or admixed. (ii) Current version: STRUCTURE 2.3.4.	http://pritch.bsd.uchicago.edu/software/structure2_2.html	Pritchard et al. [106] Falush et al. [107] Hubisz et al. [108]

fastSTRUCTURE	SNP	(i) An algorithm for inferring population structure from large SNP genotype data. (ii) It is based on a variational Bayesian framework for posterior inference and is written in Python2.x.	http://rajanil.github.io/fastStructure/	Raj et al. [109]

ADMIXTURE	SNP	(i) ADMIXTURE is a program for maximum likelihood estimation of individual ancestries from multilocus SNP genotype datasets. (ii) It uses the same statistical model as STRUCTURE but calculates estimates much more rapidly using a fast numerical optimization algorithm. (iii) Current version: ADMIXTURE 1.23.	https://www.genetics.ucla.edu/software/admixture/	Alexander et al. [110]

fineSTRUCTURE	Sequencing data	(i) A fast and powerful algorithm for identifying population structure using dense sequencing data. (ii) Current version: FineStructure 0.0.2.	http://paintmychromosomes.com/	Lawson et al. [111]

POPGENE	Use the dominant, codominant, and quantitative data for population genetic analysis	(i) Used to calculate gene and genotype frequency, allele number, effective allele number, polymorphic loci, gene diversity, observed and expected heterozygosity, Shannon index, homogeneity test, *F*-statistics, gene flow, genetic distance, dendrogram, neutrality test, and so forth. (ii) Current version: POPGENE version 1.32,	https://www.ualberta.ca/~fyeh/popgene.html	Francis et al. [112]

GENEPOP	Haploid or diploid data	(i) Used to compute exact tests or their unbiased estimation for Hardy-Weinberg equilibrium, population differentiation, and two-locus genotypic disequilibrium. (ii) It converts the input GENEPOP file to formats used by other popular programs, like BIOSYS [113], DIPLOIDL [114], and LINKDOS [115], thereby allowing communication between them. (iii) Current version: GENEPOP 4.2,	http://genepop.curtin.edu.au/	Raymond and Rousset [116]

GenAIEx	Codominant, haploid, and binary genetic data. It accommodates the full range of genetic markers available, including allozymes, SSRs, SNPs, AFLP, and other multilocus markers, as well as DNA sequences	(i) GenAIEx runs within Microsoft Excel enabling population genetic analysis of codominant, haploid, and binary data. Used to compute allele frequency-based analyses including heterozygosity, *F*-statistics, Nei's genetic distance, population assignment, probabilities of identity, and pairwise relatedness. (ii) Used for calculating genetic distance matrices and distance based calculations including analysis of molecular variance (AMOVA) [117, 118]; principal coordinates analysis (PCA); Mantel tests [119]; 2D spatial autocorrelation analyses following Smouse and Peakall [120], Peakall et al. [121], Double et al. [122]. (iii) Current version: GenAIEx 6.5.	http://biology-assets.anu.edu.au/GenAlEx/Welcome.html	Peakall and Smouse [123]

## Abbreviations

AFLP: Amplified fragment length polymorphism
AP-PCR: Arbitrarily primed PCR
ARMS: Amplification refractory mutation system
ASAP: Arbitrary signatures from amplification
ASH: Allele-specific hybridization
ASLP: Amplified sequence length polymorphism
ASO: Allele specific oligonucleotide
CAPS: Cleaved amplification polymorphic sequence
CAS: Coupled amplification and sequencing
DAF: DNA amplification fingerprint
DGGE: Denaturing gradient gel electrophoresis
GBA: Genetic bit analysis
IRAO: Interretrotransposon amplified polymorphism
ISSR: Intersimple sequence repeats
ISTR: Inverse sequence-tagged repeats
MP-PCR: Microsatellite-primed PCR
OLA: Oligonucleotide ligation assay
RAHM: Randomly amplified hybridizing microsatellites
RAMPs: Randomly amplified microsatellite polymorphisms
RAPD: Randomly amplified polymorphic DNA
RBIP: Retrotransposon-based insertion polymorphism
REF: Restriction endonuclease fingerprinting
REMAP: Retrotransposon-microsatellite amplified polymorphism
RFLP: Restriction fragment length polymorphism
SAMPL: Selective amplification of polymorphic loci
SCAR: Sequence characterised amplification regions
SNP: Single nucleotide polymorphism
SPAR: Single primer amplification reaction
SPLAT: Single polymorphic amplification test
S-SAP: Sequence-specific amplification polymorphisms
SSCP: Single strand conformation polymorphism
SSLP: Single sequence length polymorphism
SSR: Simple sequence repeats
STMS: Sequence-tagged microsatellite site
STS: Sequence-tagged site
TGGE: Thermal gradient gel electrophoresis
VNTR: Variable number tandem repeats
RAMS: Randomly amplified microsatellites.
